# Dentigerous Cyst Associated With Impacted Inverted Mesiodens: A Report of Three Cases and a Brief Literature Review

**DOI:** 10.1155/crid/6989796

**Published:** 2025-08-03

**Authors:** Manoj Adhikari, Kanistika Jha, Aashish Shah, Meenakshi Gurung, Kavita Karmacharya, Suman Gurung

**Affiliations:** ^1^Nepalese Army Institute of Health Sciences, College of Medicine, Kathmandu, Nepal; ^2^College of Medical Sciences, Bharatpur, Chitwan, Nepal

**Keywords:** case report, dentigerous cyst, impacted tooth, odontogenic cyst, supernumerary tooth

## Abstract

**Introduction:** Dentigerous cysts are the second most prevalent type of odontogenic cyst, following radicular cysts, and are primarily associated with impacted teeth. These cysts are most commonly associated with impacted third molars and canines. Notably, approximately 5% of dentigerous cysts are found in conjunction with supernumerary teeth, predominantly mesiodens. Between 1964 and April 2025, only about 51 cases of dentigerous cysts related to impacted mesiodens have been reported in the literature. This article presents three additional cases, accompanied by a concise literature review.

**Case Presentation:** This report outlines three clinical cases managed in the Oral and Maxillofacial Surgery Outpatient Department. The first case concerns a 31-year-old male who presented with a cystic swelling in the labial vestibule of the maxilla, which had persisted for 2 months. The second case involved a 25-year-old male who reported swelling in the anterior hard palate, which had been present for 6 months. The third case featured a 22-year-old male who complained of pain associated with the upper left central incisor for 1 month. Comprehensive clinical examinations and radiographic evaluations in all cases revealed cystic lesions associated with impacted inverted mesiodens. Surgical intervention was performed through enucleation of the cysts and extraction of the impacted mesiodens. Histopathological analysis confirmed the diagnosis of dentigerous cysts. Postoperative recovery was uneventful for all patients, with no complications recorded during a 1-year follow-up.

**Discussion:** Radiographically, dentigerous cysts typically appear as unilocular radiolucent lesions surrounding the crown of an impacted tooth, with the cyst attached at the cementoenamel junction. The standard treatment approach involves enucleation of the cyst and extraction of the associated impacted tooth.

**Conclusion:** Dentigerous cysts associated with impacted inverted mesiodens should be considered in the differential diagnosis of anterior maxillary swelling. Timely diagnosis and surgical intervention are critical for achieving optimal patient outcomes and mitigating potential complications.

## 1. Introduction

Cysts frequently occur in the oral and maxillofacial regions [1]. They are broadly classified into odontogenic and nonodontogenic categories [1]. Odontogenic cysts are histologically similar to normal odontogenic structures [1]. Dentigerous cysts are the most common type of developmental odontogenic cyst [2, 3]. They are the second most common odontogenic cysts, following radicular cysts [2, 4]. Dentigerous cysts form due to the expansion of the dental follicle, resulting from the accumulation of fluid between the crown of an impacted tooth and the reduced enamel epithelium [5, 6]. The third molars are the most frequently impacted teeth, followed by the canines [7]. Dentigerous cysts are most commonly associated with impacted mandibular third molars, followed by maxillary canines [5, 8]. They are rarely found in association with impacted supernumerary teeth [5, 6].

Only 5% of all dentigerous cysts are associated with impacted supernumerary teeth, mostly being impacted mesiodens [3, 4, 6]. The prevalence of mesiodens in the general population ranges from 0.15% to 1.9% [4, 9]. Mesiodens can cause various complications, including midline diastema, impaction, delayed eruption, or altered positioning of the permanent central incisors, crowding, esthetic issues, and the formation of dentigerous cysts [10, 11]. This, in turn, may lead to bone destruction, displacement of adjacent teeth, root resorption, and the formation of an oroantral fistula [10, 11].

Between 1964 and April 2025, approximately 51 cases of dentigerous cysts associated with impacted mesiodens have been documented in the English-language literature, as summarized in Table 1. The present report describes three additional cases, along with a brief review of the relevant literature. Notably, this represents the first reported cases from Nepal. We present three patients, a 31-year-old, a 25-year-old, and a 22-year-old male, diagnosed with dentigerous cysts associated with impacted inverted mesiodens. These cases were successfully managed through enucleation of the cysts and removal of the impacted mesiodens, with no complications observed during 1 year of follow-up.

## 2. Case Presentation

### 2.1. Case 1

A 31-year-old male presented to our Oral and Maxillofacial Surgery (OMFS) Outpatient Department (OPD) with a chief complaint of swelling in the anterior region of the upper jaw, which had persisted for the past 2 months. The swelling was painless, slowly increasing in size, and unaccompanied by discomfort. Past medical history revealed that the patient had an atrial septal defect, for which he had undergone surgical treatment by a cardiothoracic surgeon. He had been on medication for 6 months postoperatively.

**Figure 1:**
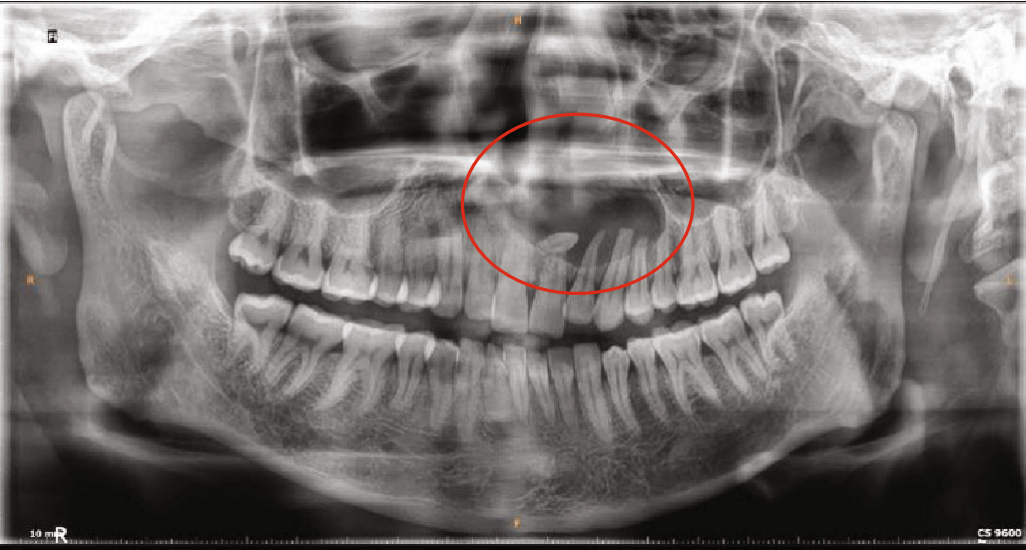
Orthopantomogram of Case 1 illustrating a radiolucent lesion in the anterior maxilla associated with an impacted inverted mesiodens.

**Figure 2:**
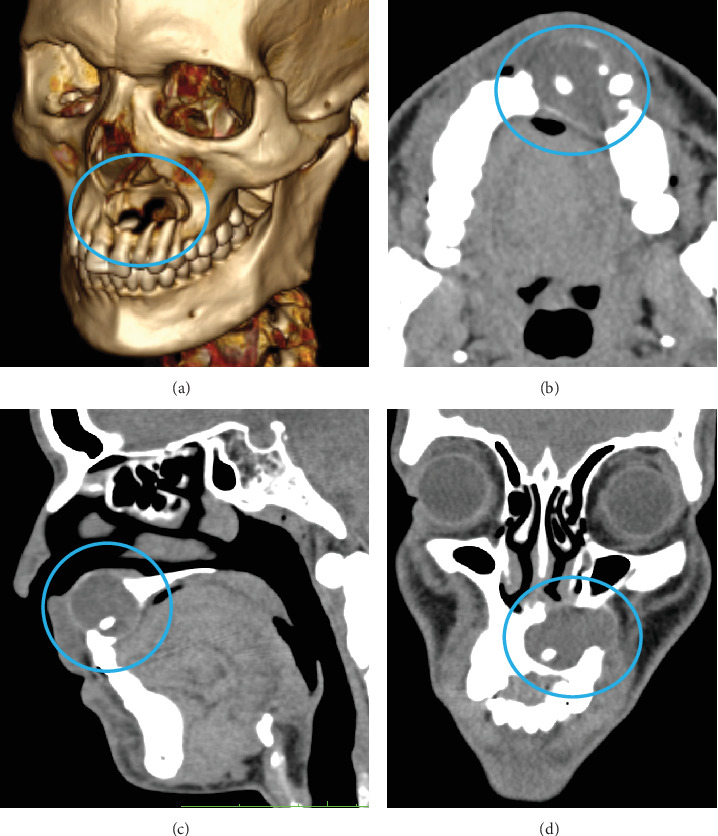
Noncontrast computed tomography images of Case 1 demonstrating a lytic lesion in the anterior maxilla associated with an impacted inverted mesiodens, with complete resorption of the labial cortical plate, nasal floor, and palatal bone. (a) Volume rendering technique image. (b) Axial section. (c) Sagittal section. (d) Coronal section.

Clinical examination revealed a mild, cystic swelling in the maxillary anterior labial vestibule, with no missing teeth. Aspiration of the lesion revealed dark brown fluid. Orthopantomogram (OPG) imaging demonstrated a well-defined, oval-shaped radiolucency, extending from the right central incisor to the left first premolar, encompassing an impacted, inverted mesiodens (Figure 1). Noncontrast computed tomography (CT) (NCCT) of the face showed a lytic lesion measuring 3.8 cm × 2.1 cm × 1.9 cm in the anterior maxilla, extending from the right central incisor to the left first premolar. The lesion had caused complete resorption of the labial cortical plate, nasal floor, and palatal bone of the anterior maxilla. An impacted inverted mesiodens with a cone-shaped crown and short root appeared as a hyperdense structure within the lytic lesion. Despite the involvement of adjacent tooth roots within the lesion, no signs of resorption were observed (Figure 2).

### 2.2. Case 2

**Figure 3:**
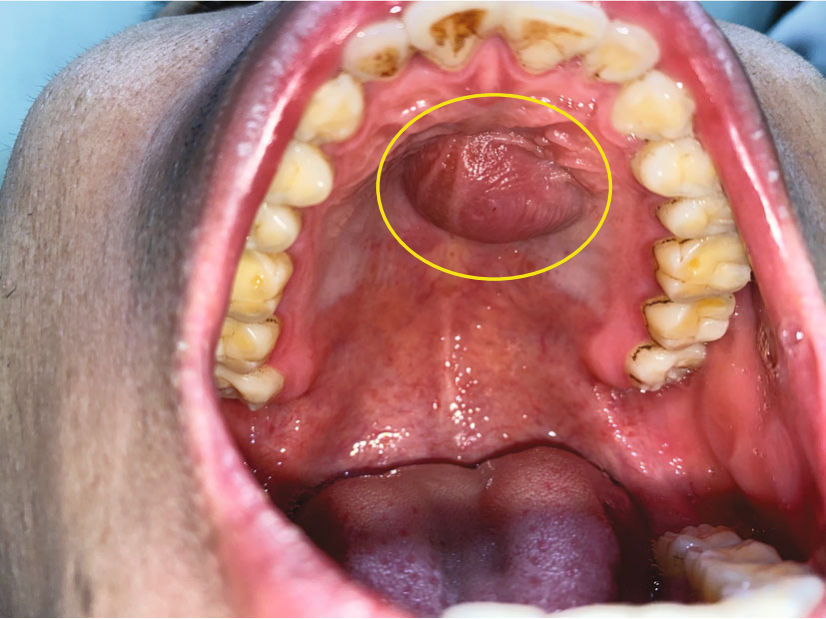
Preoperative clinical image of Case 2 displaying a cystic lesion in the hard palate.

**Figure 4:**
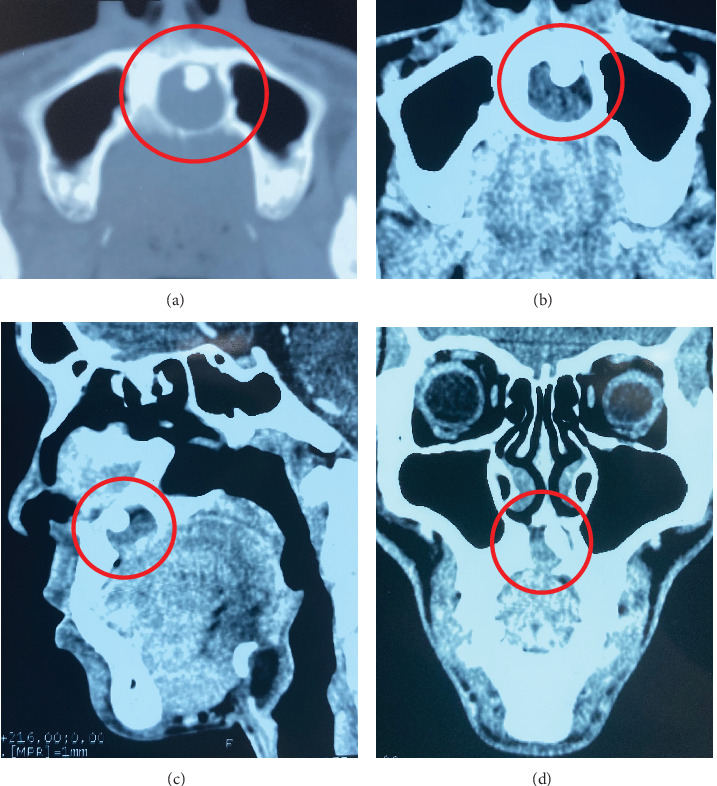
Noncontrast computed tomography (NCCT) of Case 2 reveals an expansile, nonenhancing lytic lesion containing an unerupted inverted mesiodens within the hard palate. (a) Axial section, bony window. (b) Axial section, soft tissue window. (c) Sagittal section, soft tissue window. (d) Coronal section, soft tissue window.

A 25-year-old male presented to the OMFS OPD with a chief complaint of swelling in the anterior region of the hard palate persisting for 6 months. The swelling had gradually increased in size and was associated with pain for the preceding 2 weeks. Clinical examination revealed a cystic swelling in the anterior hard palate, with no missing teeth. Aspiration of the lesion yielded pus (Figure 3). NCCT of the face revealed a well-defined, nonenhancing, lobulated expansile lesion measuring 1.8 cm × 1.9 cm × 1.5 cm within the anterior hard palate. The lesion demonstrated thin walls with areas of discontinuity. Superiorly, it was protruding toward the nasal cavity, while inferiorly, it extended into the oral cavity. An unerupted, inverted mesiodens was observed in the anterior aspect of the lesion (Figure 4).

### 2.3. Case 3

**Figure 5:**
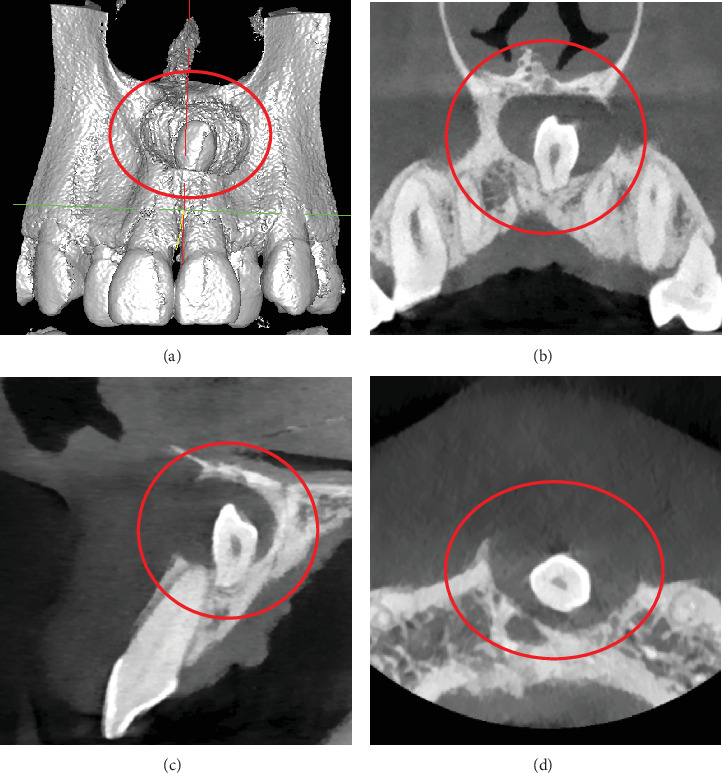
Cone beam computed tomography (CBCT) of Case 3 reveals a lytic lesion housing an unerupted inverted mesiodens within the premaxilla. (a) Three-dimensional image. (b) Coronal section. (c) Sagittal section. (d) Axial section.

A 22-year-old male presented to the OMFS OPD with a chief complaint of swelling in the maxillary labial vestibule that had persisted for 1 month. Clinical examination revealed a cystic swelling localized in the anterior labial vestibule of the maxilla, with no missing teeth noted. Aspiration of the lesion yielded a straw-colored fluid. Cone beam computed tomography (CBCT) of the facial region demonstrated a well-defined, lytic lesion measuring 1.5 cm × 1.2 cm × 1.0 cm within the premaxilla. The lesion exhibited complete resorption of the labial cortical plate, and an unerupted, inverted mesiodens was identified within the lesion (Figure 5).

In all three instances, the patients' medical, familial, and psychosocial histories were unremarkable, showing no evidence of genetic abnormalities. Additionally, routine blood investigations, chest x-rays, and electrocardiograms (ECGs) were conducted, all of which yielded results within normal limits. Based on the clinical and radiological findings, a provisional diagnosis of a dentigerous cyst associated with an impacted inverted mesiodens was made. Surgical enucleation of the cystic lesion and extraction of the impacted inverted mesiodens under general anesthesia were planned.

Under aseptic conditions, the patient was placed in a supine position, and orotracheal intubation was done. A throat pack was placed.

**Figure 6:**
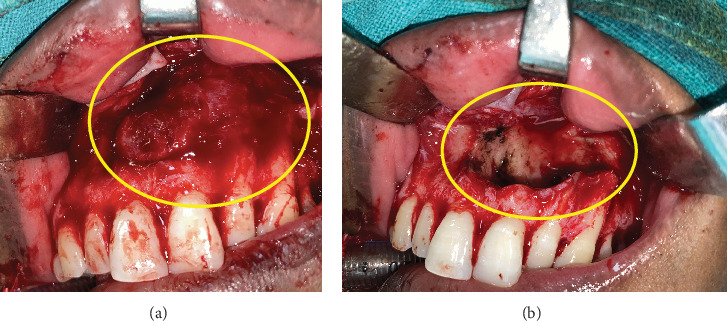
Intraoperative view of Case 1 showing (a) the cystic lesion in the anterior maxilla and (b) the bony cavity following enucleation of the cystic lesion.

In Case 1, a crevicular incision was made intraorally, extending from the right canine to the left first molar. A mucoperiosteal flap was raised, revealing the cystic lesion. Complete resorption of the labial cortical plate, nasal floor, and palatal bone of the anterior maxilla was noted. However, the soft tissues lining the nasal floor, palate, and labial vestibule were intact. During surgery, care was taken to avoid breaching these soft tissues (Figure 6).

**Figure 7:**
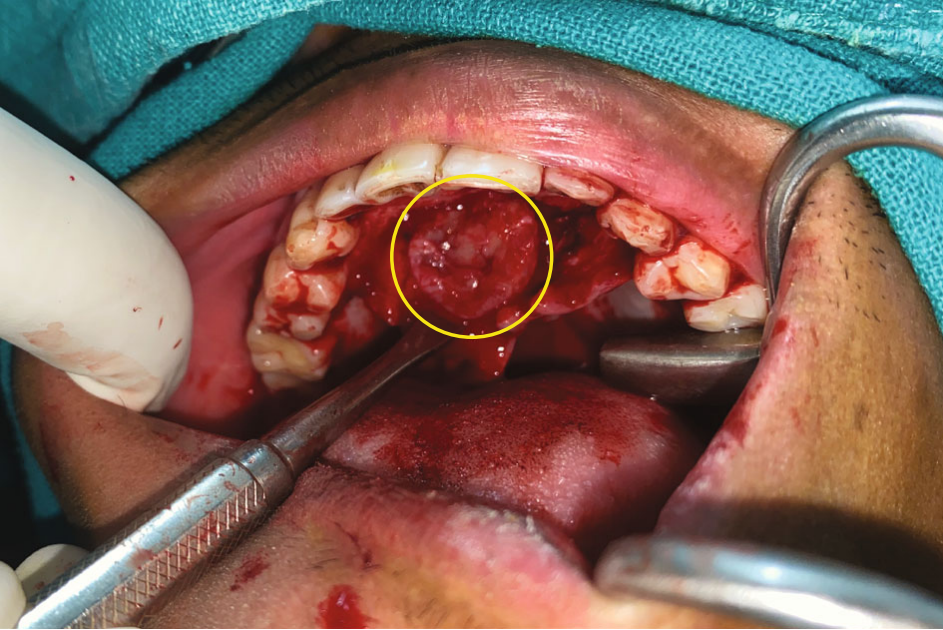
Intraoperative view of Case 2 exhibiting the cystic lesion in the hard palate.

In Case 2, a crevicular incision was made along the palatal region, extending from the right canine to the left canine. A mucoperiosteal flap was raised, exposing the cystic lesion. The palatal bone was completely resorbed, but the soft tissues lining the nasal floor were intact. Care was taken during the procedure to avoid breaching these soft tissues (Figure 7).

**Figure 8:**
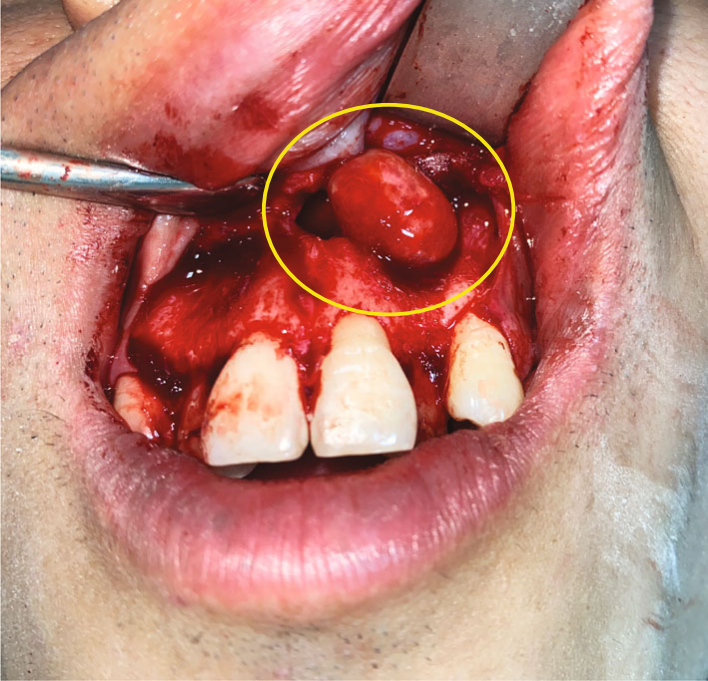
Intraoperative view of Case 3 displaying a cystic lesion in the anterior maxilla.

In Case 3, a crevicular incision was performed intraorally, extending from the right lateral incisor to the left lateral incisor. A mucoperiosteal flap was elevated, which exposed the cystic lesion. Notable complete resorption of the labial cortical plate was observed; however, the soft tissues lining the labial vestibule remained intact. Throughout the surgical procedure, precautions were taken to avoid compromising these soft tissues (Figure 8).

In all three cases, the cystic lesions, along with the impacted inverted mesiodens, were enucleated and sent for histopathological examination. Hemostasis was achieved, and primary closure was done. The throat pack was removed, and the patient was extubated.

Postoperatively, the patient was prescribed antibiotics and analgesics, with daily dressing done for 5 days.

## 3. Histopathological Examination

**Figure 9:**
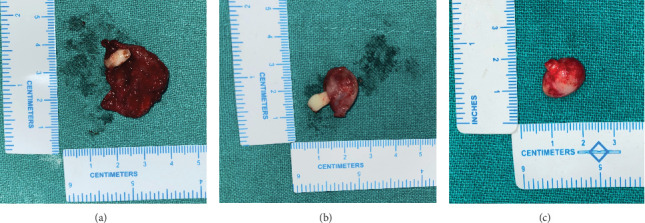
Intraoperative view of the specimen prepared for histopathological examination, consisting of the cystic lesion lining and the impacted mesiodens. (a) Case 1, (b) Case 2, and (c) Case 3.

*Gross findings:* A singular globular tissue mass, accompanied by a single tooth, was observed in all three cases. In Case 1, the specimen measured 2.5 cm × 2.5 cm × 1 cm; in Case 2, it measured 1.5 cm × 1 cm × 1 cm; and in Case 3, it measured 1.5 cm × 1 cm × 0.5 cm (Figure 9).

**Figure 10:**
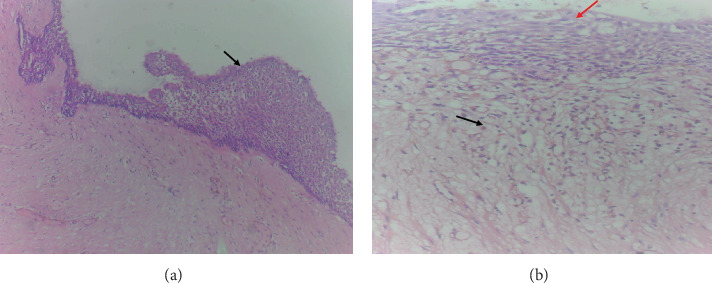
Microscopic views of Case 1. (a) 10× magnification revealing the stratified squamous epithelial lining of the cyst wall with variable thickness (black arrow). (b) 40× magnification, with the red arrow indicating the stratified squamous epithelial lining of the cyst wall, and the black arrow denoting dense inflammatory infiltrates composed of lymphocytes, plasma cells, and neutrophils.

**Figure 11:**
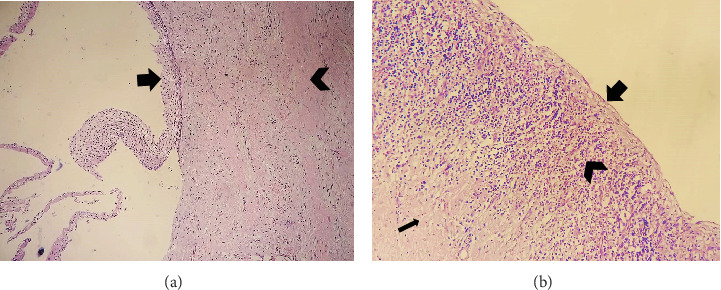
Microscopic views of the lesion in Case 2. (a) 10× magnification displaying a cyst wall lined by stratified squamous epithelium (arrow) with underlying subepithelial fibrous connective tissue (chevron). (b) 40× magnification showing an absence of epithelial lining (thick arrow), with underlying subepithelial dense mixed inflammatory infiltrates comprising lymphocytes, plasma cells, neutrophils, and eosinophils (chevron) alongside fibrous connective tissue (thin arrow).

**Figure 12:**
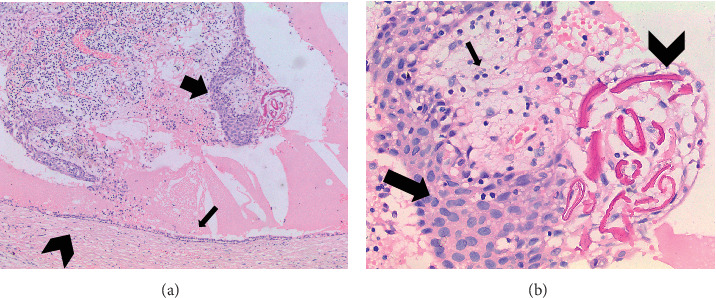
Microscopic views of the lesion in Case 3. (a) 10× magnification illustrating a cyst wall lined by flattened epithelial cells (thin arrow) and a stripped stratified squamous epithelium (thick arrow) with underlying subepithelial fibrous connective tissue (thick chevron). (b) 40× magnification displaying the stratified squamous epithelial lining (arrow) along with Rushton bodies (chevron) and chronic inflammatory cells (thin arrow).

*Microscopic findings:* Histological sections revealed a cyst wall lined by stratified squamous epithelium with a focal area showing stripped-off squamous epithelium. The underlying subepithelium consisted of fibrous connective tissue with mixed inflammatory infiltrates comprising lymphocytes, plasma cells, neutrophils, and eosinophils (Figures 10, 11, and 12).

Based on clinical, radiological, surgical, and histopathological findings, the final diagnosis was confirmed as “dentigerous cyst associated with an impacted inverted mesiodens.”

The patients were followed up regularly. During follow-up visits, recurrence, oroantral communication, and discoloration of adjacent teeth were checked. No complications were noted, and the patient remained asymptomatic during the 1-year follow-up.

## 4. Discussion

The term “dentigerous” refers to “containing tooth” or “tooth bearing,” a characteristic feature of a dentigerous cyst [8, 11]. These cysts account for 16.6% of all jaw cysts [9, 20]. Approximately 95% of dentigerous cysts are associated with permanent teeth, while only about 5% are associated with supernumerary teeth [6, 9]. The premaxilla is the most common site for supernumerary teeth [3]. In the maxillary midline, the supernumerary tooth is referred to as a mesiodens due to its location in the center of the upper jaw [8]. Mesiodens is the most common type of supernumerary tooth, located between the two maxillary permanent central incisors [4, 5].

The most prevalent disorder of odontogenesis is the formation of supernumerary teeth [10, 44]. The exact etiology of supernumerary teeth remains unknown; however, it is hypothesized to result from disruptions in odontogenesis, splitting of the enamel organ, uncontrolled cell proliferation, hyperactivity of the dental lamina, genetic mutations, or environmental factors [10, 11].

Supernumerary teeth can be associated with various developmental disorders, including cleidocranial dysplasia, cleft lip and palate, Gardner's syndrome, and Down syndrome [10, 44]. Dentigerous cysts are typically solitary, although multiple cysts can occur in syndromic cases, such as basal cell nevus syndrome, Gardner's syndrome, Maroteaux–Lamy's syndrome, and mucopolysaccharidosis [11]. In the present cases, the lesions were solitary and unrelated to any syndrome.

Supernumerary teeth can be classified into four types: conical, tuberculate, supplemental, and odontoma [10, 44]. In the present cases, the supernumerary teeth were conical.

The association between dentigerous cysts and supernumerary teeth was first described by Pitts in 1924 [6, 11]. In 1931, Stafne found an incidence of 5.5% in a study of 200 supernumerary teeth in 180 patients [5, 8].

Mesiodens can present as a single or multiple teeth [4, 28]. They are usually impacted [5, 28]. They are rarely associated with dentigerous cysts [4, 33]. Mesiodens typically have a cone-shaped crown and a short root [4, 28]. Based on the studies summarized in Table 1, the gender consisted of 37 males and 14 females, indicating a higher prevalence among males, with a male-to-female ratio of 2.6:1. In the present cases, single inverted mesiodens associated with dentigerous cysts were identified in male patients, consistent with previous literature.

Dentigerous cysts commonly affect individuals in their second and third decades of life, although they can occur between the ages of 8 and 71 years [8, 20]. Dentigerous cysts associated with supernumerary teeth are usually present in the first four decades of life [11, 33]. According to the studies summarized in Table 1, the age of patients affected by dentigerous cysts associated with impacted inverted mesiodens ranges from 8 to 71 years, with a mean age of 32 years. In the present cases, the patients were 31, 25, and 22 years of age, respectively, which is consistent with the previously reported age range.

Clinically, dentigerous cysts are often associated with missing teeth and can present as a bony hard swelling, occasionally resulting in facial asymmetry and rarely causing pathological fractures [8, 33]. These cysts can cause adjacent bone resorption due to stimuli such as infection, trauma, inflammation, or metabolic changes [3]. In the present cases, fluctuant cystic swellings were present in the anterior maxillary labial vestibule and the anterior part of the hard palate, with complete resorption of the adjacent bone. No teeth were missing, as the cysts were associated with supernumerary teeth. The cysts were located in the anterior maxilla and the anterior part of the hard palate, with details revealed only through computed tomographic images.

Radiographically, dentigerous cysts appear as unilocular radiolucencies with well-defined sclerotic margins associated with the crown of an impacted tooth [28, 33]. When infected, the cyst exhibits ill-defined margins [8, 33]. Occasionally, a large dentigerous cyst may demonstrate a multilocular appearance [11]. A key feature of dentigerous cysts is their attachment at the cementoenamel junction of the impacted tooth [10, 28]. Radiographically, dentigerous cysts can be classified into three varieties: (i) the central variety, in which the crown of the tooth is located at the center of the cystic lumen; (ii) the lateral variety, in which the cyst is situated laterally along the root of the tooth; and (iii) the circumferential variety, characterized by the cyst surrounding the crown while extending along the root surface [11]. In the cases presented, all lesions were unilocular and classified as the central variety, consistent with the existing literature.

The lesions, such as adenomatoid odontogenic tumor (AOT), odontogenic keratocysts (OKCs), hyperplastic dental follicles, and unicystic ameloblastomas, may also be associated with impacted teeth [11, 20, 45]. These lesions should be considered in the differential diagnosis.

In the context of AOT, it is also known as “two-thirds tumor,” that is, it has been observed that approximately two-thirds of affected individuals are female, two-thirds of cases occur during the second decade of life, and two-thirds of the tumors are located in the anterior region of the maxilla [46]. Additionally, two-thirds of AOT cases are associated with dentigerous cysts, and a similar proportion involves unerupted permanent canines [46]. Radiographically, the associated radiolucency extends apically beyond the cementoenamel junction [20, 31]. Microscopically, AOT is characterized by solid nodules composed of polygonal, cuboidal, or spindle-shaped odontogenic epithelial cells [46]. These cells organize into nests, duct-like spaces, rosette-like structures, and strands, presenting a trabecular and cribriform architecture within a mature connective tissue stroma, which is encapsulated by fibrous tissue [46]. Notably, intercellular eosinophilic amorphous material and varying quantities of calcified material are present in the majority of lesions [46].

OKCs are associated with impacted teeth in approximately 40% of cases [20, 31]. The lumen of OKCs is characterized by the presence of keratinous material, and it is lined by a parakeratinized epithelium [31]. Compared to other odontogenic cysts, OKCs exhibit more aggressive growth patterns [31]. Radiographically, these lesions may present as a multilocular appearance with undulating borders [31].

Ameloblastoma is a slow-growing yet locally invasive tumor characterized by painless swelling of the mandible or maxilla [47]. Its growth occurs primarily in the buccolingual direction, leading to significant expansion that can result in malocclusion, facial deformity, soft tissue invasion, and loosening of adjacent teeth [47]. The most common age of presentation is between 30 and 60 years, with the mandible being the most frequently affected site [47]. The unicystic variant of ameloblastoma is more commonly observed in the pediatric population and is thought to arise from pre-existing dentigerous cysts or dental follicles, given its frequent association with unerupted teeth [47]. Radiographically, conventional ameloblastoma is characterized by a classic “soap bubble” appearance, while unicystic ameloblastoma presents as a lytic lesion with scalloped margins [47]. CT scans typically reveal well-defined, unilocular or multilocular lytic expansile lesions [47]. The unicystic form is often diagnosed only after histopathological examination, as it can resemble an odontogenic cyst both clinically and radiologically [47]. Histologically, ameloblastoma is composed of two distinct cell types: basally located “basal cells” resembling ameloblasts and suprabasal “epithelial cells” that resemble stellate reticulum [47]. The basal cells exhibit hyperchromasia, a columnar shape, palisaded arrangement, vacuolated cytoplasm, and displaced nuclei away from the basement membrane [47]. In contrast, the epithelial cells demonstrate a bland cytological appearance, with sparse mitotic figures indicative of their slow rate of growth [47].

When the radiolucent space around an impacted tooth exceeds 5 mm, it is considered a dentigerous cyst; smaller spaces are classified as dental follicles [10, 33].

Although impacted teeth can be detected on plain radiographs, detailed pathological information is often lacking [2, 8]. CT scans are preferred over plain radiographs, as they provide more accurate information regarding the size, extent, and location of the pathology [2, 3]. CBCT is an effective technique for the accurate localization of pathologic lesions, significantly aiding in surgical planning [44]. In comparison to NCCT, CBCT offers the advantage of reduced ionizing radiation exposure while still delivering sufficient information for the assessment of dental pathologies [44]. Nevertheless, it is important to note that the overall image quality achieved with NCCT is superior to that of CBCT, primarily due to the inherent limitations of CBCT in differentiating soft tissue structures [44].

In the current cases, CBCT was conducted in one instance, while NCCT scans were performed in two instances, aligning with the existing literature.

Diagnosing dentigerous cysts associated with impacted supernumerary teeth can pose significant clinical challenges, particularly in the absence of an identifiable clinically missing tooth. However, imaging modalities are crucial in this context, as they provide valuable information regarding the presence of impacted teeth and delineate the extent of the associated pathology, thereby facilitating accurate diagnosis.

The treatment of choice for dentigerous cysts associated with supernumerary teeth is the cyst's enucleation and extraction of the impacted tooth [2, 20]. For large cysts, marsupialization followed by enucleation and extraction is recommended [2, 20]. In the present cases, enucleation of the cyst and extraction of the impacted inverted mesiodens were performed as described in the literature.

Histologically, dentigerous cysts are lined by nonkeratinized stratified squamous epithelium, surrounded by a thin connective tissue wall containing odontogenic epithelial rests [2, 11]. The histological findings in the present cases were consistent with these characteristics, aligning with the literature.

Residual remnants of dentigerous cysts following surgical intervention may lead to recurrence and possess the capacity for neoplastic transformation [48]. Neoplastic transformation associated with odontogenic cysts accounts for less than 3% of cases [49]. Odontogenic cysts can undergo malignant transformation into various neoplasms, including central mucoepidermoid carcinoma, primary intraosseous squamous cell carcinoma, ameloblastoma, squamous cell carcinoma, and ameloblastic carcinoma [49]. These neoplastic lesions can originate from a range of cyst types, such as dentigerous cysts, OKCs, calcifying odontogenic cysts, glandular odontogenic cysts, residual cysts, radicular cysts, follicular cysts, and unspecified odontogenic cysts [49]. Therefore, the surgical excision must target the complete removal of all pathological tissue to mitigate the risks of recurrence and neoplastic transformation.

## 5. Conclusion

Dentigerous cysts associated with impacted inverted mesiodens should be included in the differential diagnosis for swelling in the anterior maxillary vestibule or the anterior hard palate. Prompt diagnosis and surgical intervention are essential to achieving optimal patient outcomes and preventing potential complications.

## Figures and Tables

**Table 1:** Overview of dentigerous cysts associated with impacted mesiodens.

**SN**	**Study**	**Country**	**No. of cases**	**Age (years)/sex**
1	Present case	Nepal	3	22/M, 25/M, 31/M
2	Khandelwal et al. [12]	India	1	35/M
3	Jadaun et al. [13]	India	1	26/M
4	Abbasi et al. [14]	Iran	1	30/M
5	Hutomo et al. [15]	Indonesia	1	33/F
6	Rivas et al. [16]	Chile	1	43/M
7	Baranwal et al. [17]	India	1	18/M
8	Wu et al. [18]	Taiwan	1	52/F
9	Nair et al. [19]	India	1	32/M
10	Reddy et al. [20]	India	1	23/M
11	Mostafazadeh et al. [21]	Iran	1	45/F
12	Mishra et al. [22]	India	1	30/M
13	Supreetha et al. [23]	India	1	32/M
14	Soumithran et al. [24]	India	1	8/M
15	Deepak et al. [8]	India	1	32/M
16	Narsapur et al. [25]	India	1	40/M
17	Mahmud et al. [26]	India	1	56/F
18	Hasan et al. [11]	India	1	32/M
19	Kim and Mun [3]	Korea	1	35/M
20	Shah et al. [27]	India	1	18/M
21	Patel et al. [28]	India	1	30/M
22	Ramaswamy et al. [29]	India	1	45/M
23	Byatnal et al. [30]	India	1	13/M
24	Khambete et al. [2]	India	2	55/M, 46/M
25	Jiang et al. [31]	China	4	55/F, 46/M, 53/M, 23/M
26	Ravi et al. [32]	India	1	31/M
27	Kalasakar et al. [10]	India	2	Both 12/M
28	Hosseini et al. [33]	Iran	1	18/F
29	Anand Kumar et al. [34]	India	1	12/M
30	Mehta et al. [35]	India	1	22/M
31	Deepak et al. [9]	India	1	12/F
32	Yekyung [36]	South Korea	1	8/M
33	Khan et al. [37]	Bangladesh	1	24/M
34	Dinkar et al. [4]	India	1	14/F
35	Scolozzi et al. [5]	Switzerland	1	42/M
36	Hashida et al. [38]	Japan	1	37/M
37	Som et al. [39]	United States	1	39/F
38	Awang et al. [40]	Ireland	2	34/M, 24/F
39	Lustmann and Bodner [41]	Israel	6	9/F, 12/M, 37/M, 38/M, 68/F, 71/F
40	Papadopoulos [42]	Libya	1	40/F
41	Ames [43]	United States	1	39/M

Abbreviations: F, female; M, male.

## Data Availability

Data sharing is not applicable to this article as no new data were created or analyzed in this study.

## General Statement

*Patient Perspective*. The patients expressed high satisfaction with the successful treatment outcome, achieved without complications.
